# Reconstruction of the upper gastrointestinal tract using intra-thoracic Roux-en-Y technique after caustic agent ingestion: a case report from Aleppo, Syria

**DOI:** 10.1186/s13019-023-02237-x

**Published:** 2023-04-11

**Authors:** Samer Alhames, Mamdouh Alkhaled, Mike Ghabally

**Affiliations:** 1 Fellow at The French College of Thoracic and Cardiovascular Surgery, Chief of Thoracic Surgery Department at Saint Louis Hospital, Aleppo, Syria; 2 Faculty of Medicine, Department of Internal Medicine, Division of Gastroenterology, University of Aleppo, Aleppo, Syria; 3 Faculty of Medicine, Department of Internal Medicine, Division of Cardiology, University of Aleppo, Aleppo, Syria

**Keywords:** Corrosive ingestion, Esophagus stricture, Pyloric stenosis, Roux-en-Y, Gastric pull-up, Case Report

## Abstract

**Background:**

Caustic substance ingestion is a high-risk medical emergency associated with high mortality and morbidity. To date, there are several treatment options with no standard method of care.

**Case presentation:**

We report a case of a corrosive agent ingestion complicated with third-degree burns and severe stenosis of the esophagus and gastric outlet. After failure of conservative treatment, the patient underwent jejunostomy placement for nutritional support followed by transhiatal esophagectomy with gastric pull-up and intra-thoracic Roux-en-Y gastroenterostomy with good outcomes. The patient recovered from the procedure and has been tolerating oral intake very well with significant weight gain.

**Conclusion:**

We put a new technique for treating severe gastrointestinal injuries caused by corrosive agent ingestion that resulted in both esophageal and gastric outlet strictures. These rare complex cases requires difficult treatment decisions. We believe that this technique provides many benefits for such cases and might be a feasible alternative for colon interposition.

## Background

Caustic substance ingestion is a medical emergency associated with high mortality and morbidity [1, 2].

We present a new technique for treating patients with severe long esophageal stenosis associated with gastric outlet obstruction. This technique has not been previously reported and we believe that it can provide an effective alternative surgical technique with a good quality of life.

The management of such cases remains challenging and there is no gold standard surgical approach [3]. The type of surgery should be decided case-by-case based on the case presentation and the type of damage.

## Case presentation

A 17-year old female presented with coffee-ground emesis and epigastric pain following ingestion of sodium hydroxide in a suicide attempt. Urgent Endoscopy demonstrated third - degree burns in the esophagus, stomach and duodenal bulb. She was treated conservatively in another institution and was discharged in a stable condition. Two months later, she started experiencing nausea, vomiting, dysphagia to both solids and liquids with a significant malnutrition and weight loss (67 to 35 kg). Barium swallow esophageogram demonstrated a severe long stricture in the middle and distal thirds of esophagus with contrast retention in the stomach indicating severe pyloric stenosis.

The patient underwent several esophageal dilation courses without symptomatic improvement. Thus, she underwent jejunostomy placement to improve the general status before the surgical intervention.

**Fig. 1:**
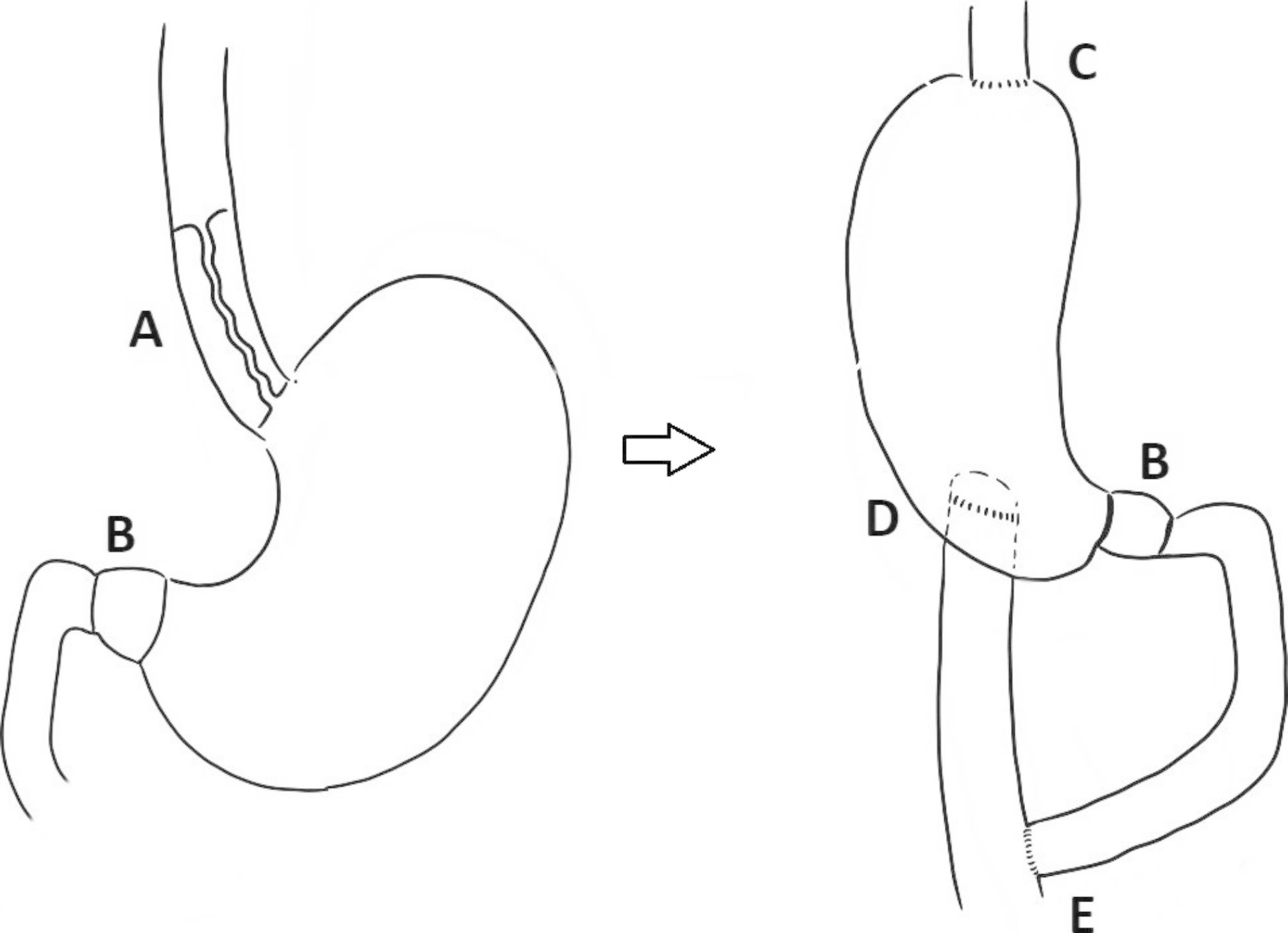
Line Drawing of the caustic injury and the surgical technique plan demonstrating a severe long stenosis of middle and distal thirds of the esophagus (**A**), pyloric total occlusion (**B**) end-to-side esophago-gastric anastomosis (**C**), side-to-side gastro-jejunal anastomosis (**D**) and end-to-side jejuno-jejunal anastomosis (**E**); the roux limb measuring 50 cm and the biliopancreatic limb measuring 70 cm in length

The patient was referred to our institution at this point for surgical consultation. Given her age and the localization and number of strictures, our surgeon decided a new technique to treat this rare case. This technique composed of laparotomy and cervicotomy, subtotal esophagectomy, gastric pull-up, gastro-jejunal anastomosis as a roux-en-Y technique and cervical esophago-gastric anastomosis. There was three anastomosis, esophago-gastric (end-to-side), gastro-jejunal (side-to-side) and jejuno-jejunal (end-to-side). All anastomosis were hand sewn (Figure 1)

**Fig. 2:**
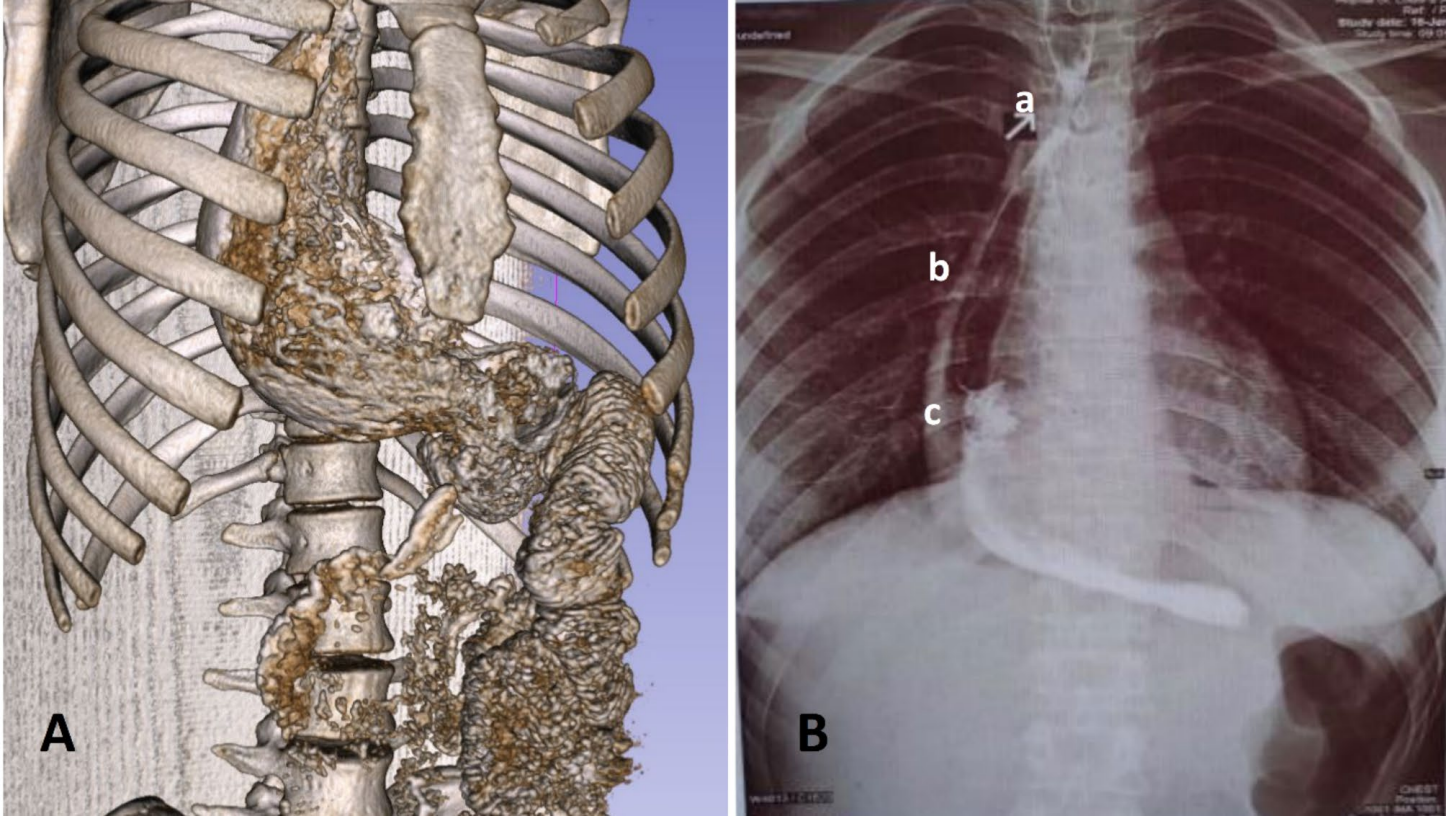
**A**: 3-dimentional view of the multi-Slice computerized tomography scan with barium swallowing after the new procedure demonstrating a good passage of contrast agent through the anastomosis. **B**: Chest X-ray with barium swallowing demonstrates a stricture in the esophago-gastric anastomosis (a), the pulled-up stomach (b) and the gastro-jejunal anastomosis (c)

On postoperative day 9, the patient was complicated with fistula presenting with food discharges in the level of the esophago-gastric anastomosis after eating against instructions. Methylene-blue dye swallowing test confirmed the diagnosis and she responded well to conservative medical therapy. On day 15, the patient was discharged in a well situation. Six weeks later, she presented with dysphagia and barium swallow study revealed a 1 cm long stenosis 20 cm from the incisors. Patient underwent several endoscopic dilations with good results. On follow up for 18 months, she was asymptomatic with well general status and significant weight gain (35 to 62Kg).

## Discussion

Corrosive ingestion mostly occurs accidently causing a wide spectrum of injuries and lifelong morbidity. The most common long-term complication is stenosis occurring in up to 90% of cases. Esophagogastroduodenoscopy is highly recommended in the first 12–48 h to evaluate the upper gastrointestinal tract damage and prognosis [1, 2].

Nonsurgical treatment should be performed before considering interventional methods except for surgical emergencies and severe complicated cases that require immediate intervention. In symptomatic patients with esophageal stenosis, esophageal dilatation is usually performed within 3 weeks, and continues for 3 months until improving the symptoms [2–3]. In cases of esophageal stenosis longer than 3 cm, multiple strictures or symptomatic patients despite appropriate endoscopic dilation, esophagus replacement is indicated [1–3] In these cases, management should be decided case-by-case as there are no established guidelines in the medical literature for the optimal surgical treatment. In our case, esophageal dilation failed and symptoms did not improve, so esophageal replacement was necessitated.

Esophageal replacement has two major types, esophagocoloplasty and gastric transposition [3]. In our case the stricter was too long and accompanied with gastric outlet obstruction, thus, gastric transposition alone was not valid.

According to the medical literature of esophageal replacement; first, in comparison with the colon, the stomach is easier to prepare and gastric vasculature is more reliable, which provides less risk of necrosis. Second, while gastric pull-up have only one anastomosis, esophagocoloplasty includes three anastomosis resulting in a higher risk of restenosis and fistula formation due to the vascularity of the colon. Furthermore, gastric pull-up has less over-all morbidity and mortality than colon interposition. However, although the stomach has a sufficient length that reaches the neck, the colon is longer and maintains the function of the stomach. Additionally, acid reflux complications are more common in gastric pull-up [3].

In our case, the patient had simultaneous injury of the esophagus and the pylorus sparking the antrum and the body of the stomach. We had two alternatives to our technique, either to perform esophagogastrectomy and coloplasty by colojejunal anastomosis (4anastomosis); or to perform coloplasty with cologastric anastomosis and gastrojejunostomy for the pyloric stenosis (4anastomosis). However, we decided to perform an esophagectomy with gastric pull-up and intrathoracic gastro-jejunal Roux-en-Y anastomosis adding an extra length to the conduit, reducing the risk of postoperative biliary reflux in addition to sparing the colon in a young adult, which is a major bonus as well [4–6]. The decision between gastric pull-up and colonic interposition is a major debate in the medical literature, and until date, there are no clear guidelines regarding the appropriate choice. Although gastric pull-up has been the predominant technique for esophageal replacement, many papers recommended that colonic interposition has a non-inferiority to gastric pull-up with a limited mortality and considerable morbidity [5]. On the other hand, despite the high anastomotic rate and reflux, trans-hiatal esophagectomy and gastric pull-up with cervical anastomosis is still considered a safe procedure that can be performed for corrosive esophageal stricture. Thus, the decision between the two procedures should be personalized and discussed case-by-case [6].

Our search through the medical literature revealed no such procedure. We believe that this technical procedure provides a life-changing alternative to traditional surgeries in patients with a combination of long esophageal and pyloric stricture. Additionally, it provides a valid option in patients with previously diseased colon especially patients with inflammatory bowel disease.

## Conclusion

We present a new technique for treating patients with long esophageal stenosis associated with gastric outlet obstruction. We believe that gastric pull-up, pyloric exclusion and intrathoracic Roux-en-Y gastroenterostomy can provide an effective alternative to traditional surgical techniques with better quality of life. However, more studies should be performed in this field to evaluate the complications and outcomes of this procedure.
